# Short sleep duration and poor sleep quality predict next-day suicidal ideation: an ecological momentary assessment study

**DOI:** 10.1017/S0033291718001009

**Published:** 2018-04-26

**Authors:** Donna L. Littlewood, Simon D. Kyle, Lesley-Anne Carter, Sarah Peters, Daniel Pratt, Patricia Gooding

**Affiliations:** 1 Division of Psychology & Mental Health, School of Health Sciences, University of Manchester, Manchester, UK; 2 Manchester Academic Health Science Centre, University of Manchester, Manchester, UK; 3 NIHR Greater Manchester Patient Safety Translational Research Centre, Manchester, UK; 4 Sleep and Circadian Neuroscience Institute, Nuffield Department of Clinical Neurosciences, University of Oxford, UK; 5 Centre for Biostatistics, School of Health Sciences, University of Manchester, Manchester, UK; 6 Manchester Centre for Health Psychology, University of Manchester, Manchester, UK

**Keywords:** Actigraphy, ecological momentary assessment, entrapment, suicide, sleep disturbance, suicidal ideation

## Abstract

**Background:**

Sleep problems are a modifiable risk factor for suicidal thoughts and behaviors. Yet, sparse research has examined temporal relationships between sleep disturbance, suicidal ideation, and psychological factors implicated in suicide, such as entrapment. This is the first in-the-moment investigation of relationships between suicidal ideation, objective and subjective sleep parameters, and perceptions of entrapment.

**Methods:**

Fifty-one participants with current suicidal ideation completed week-long ecological momentary assessments. An actigraph watch was worn for the duration of the study, which monitored total sleep time, sleep efficiency, and sleep latency. Daily sleep diaries captured subjective ratings of the same sleep parameters, with the addition of sleep quality. Suicidal ideation and entrapment were measured at six quasi-random time points each day. Multi-level random intercept models and moderation analyses were conducted to examine the links between sleep, entrapment, and suicidal ideation, adjusting for anxiety and depression severity.

**Results:**

Analyses revealed a unidirectional relationship whereby short sleep duration (both objective and subjective measures), and poor sleep quality, predicted the higher severity of next-day suicidal ideation. However, there was no significant association between daytime suicidal ideation and sleep the following night. Sleep quality moderated the relationship between pre-sleep entrapment and awakening levels of suicidal ideation.

**Conclusions:**

This is the first study to report night-to-day relationships between sleep disturbance, suicidal ideation, and entrapment. Findings suggest that sleep quality may alter the strength of the relationship between pre-sleep entrapment and awakening suicidal ideation. Clinically, results underscore the importance of assessing and treating sleep disturbance when working with those experiencing suicidal ideation.

## Introduction

The most recently released figures indicate that there were 10.4 suicides per 100 000 people in the UK (Office for National Statistics, 2017) and 13.42 suicides per 100 000 people in the USA, in 2016 (Centers for Disease Control & Prevention, 2017). Furthermore, a survey in England revealed that an estimated 20.6% of people have experienced suicidal thoughts during their lifetime, and 6.7% have previously attempted suicide (McManus *et al.* 2016). Improving the understanding of factors and mechanisms underpinning pathways to suicidal thoughts and attempts is crucial for the development of effective suicide-focused interventions.

One such factor found to be associated with increased vulnerability for suicidal ideation and behaviors is sleep disturbance (Pigeon *et al.* 2012; Malik *et al.* 2014; Littlewood *et al.* 2017). Suicidal ideation has been linked to specific types of sleep problems, such as insomnia (Zuromski *et al.* 2017) and nightmares (Nadorff *et al.* 2014; Littlewood *et al.* 2016*b*). Specific sleep parameters are also associated with increased likelihood of suicidal thoughts, attempts, and death by suicide, including short self-reported sleep duration (Gunnell *et al.* 2013; Winsler *et al.* 2015), low sleep efficiency (the proportion of time in bed spent asleep; Sabo *et al.* 1991; Bernert *et al.* 2017*b*), and poor sleep quality (Turvey *et al.* 2002; Bernert *et al.* 2014). Research has largely adopted cross-sectional designs to investigate the association between sleep disturbance and suicidal ideation. This is problematic because of the fluctuation of both sleep problems (Vallieres *et al.* 2005; Lemola *et al.* 2013) and suicidal ideation (Ben-Zeev *et al.* 2012; Kleiman *et al.* 2017). Hence, such designs fail to characterize the temporal aspects of this relationship. Temporality has been addressed by three studies that investigated the possible bidirectional associations between symptoms of sleep disturbance and suicidal or self-harmful thoughts. In a longitudinal questionnaire study, Ribeiro *et al.* (2012) observed a unidirectional relationship between self-reported insomnia symptoms at baseline and suicidal ideation 1 month later. In a more recent study, participants completed six self-report assessments of insomnia symptoms and suicidal ideation via questionnaires, every 3 days across a 15-day period (Zuromski *et al.* 2017). Insomnia symptoms predicted a subsequent increase in the frequency and intensity of suicidal ideation, but suicidal ideation failed to predict subsequent changes in insomnia symptoms. A 5-day diary study assessed bidirectional relationships between nightmares and self-harmful thoughts and behaviors in a sample of university students, whilst controlling for depressive symptoms and negative affect (Hochard *et al.* 2015). A unidirectional relationship was reported whereby the occurrence of nightmares significantly predicted post-sleep self-harming thoughts and behaviors. However, the model that examined the reverse relationship was non-significant.

Over-reliance on retrospective, self-report measures of sleep is a considerable limitation of research conducted in this area (Pigeon *et al.* 2012; Littlewood *et al.* 2017). Subjective measurement is susceptible to recall biases whereby individuals with mental health problems report greater levels of sleep disturbance when completing retrospective sleep quality questionnaires, such as the Pittsburgh Sleep Quality Index (PSQI; Buysse *et al.* 1989), in comparison with the data recorded using daily sleep diaries (Hartmann *et al.* 2015). Alternatively, objective estimates of sleep can be used to address this issue of recall bias. Relatively few studies have used objective estimates of sleep to investigate the association between sleep disturbance and suicidal ideation and behaviors. For example, studies utilizing polysomnography (PSG) have reported associations between suicidal ideation and lower sleep efficiency (Sabo *et al.* 1991; Bernert *et al.* 2017*b*); greater nocturnal awakenings (Ballard *et al.* 2016; Bernert *et al.* 2017*b*); and abnormalities in rapid-eye movement (REM) sleep, such as increased REM activity and time spent in REM sleep (Sabo *et al.* 1991; Keshavan *et al.* 1994; Agargun & Cartwright, 2003). The relationship between sleep disturbance and suicidal ideation was recently examined with wrist actigraphy, which monitors movement through accelerometry to provide an objective estimate of sleep (Bernert *et al.* 2017*a*). A sample of university students wore actigraphy watches for 7 days and completed measures of suicidal ideation at baseline, 7, and 21 days later. Analyses showed that the variability in sleep/wake patterns predicted increased suicidal ideation at 7- and 21-day follow-up, independent of depressive symptoms (Bernert *et al.* 2017*a*).

Although sleep disturbance has been associated with suicidal thoughts and behaviors, not everyone who experiences sleep disturbance will develop suicidal thoughts or attempt suicide. Consequently, it is important to understand the mechanisms by which sleep disturbance may lead to suicidal ideation and behavior. For instance, one such candidate mechanism is entrapment, which is a psychological construct that refers to the desire to escape together with the perception that escape is not possible (Gilbert & Allan, 1998). Contemporary theories of suicidal behavior posit that entrapment is a proximal factor for suicidal thoughts (Johnson *et al.* 2008; O'Connor, 2011; O'Connor & Portzky, 2018).

Preliminary evidence from three methodologically diverse studies indicates that perceptions of entrapment may be important in understanding the associations between sleep problems, and suicidal thoughts and behaviors (Littlewood *et al.* 2016*a*, *b*; Hochard *et al.* 2017). First, entrapment was shown to partially mediate the relationship between nightmares and suicidal behaviors (Littlewood *et al.* 2016*b*). Second, entrapment was found to interact with both insomnia and nightmares to predict suicidal thoughts (Hochard *et al.* 2017). Both of these studies used cross-sectional designs. Finally, participants in a qualitative study described how sleep provided a welcome escape from suicidal thoughts and mental distress, which for some also produced improved mood and coping abilities on waking (Littlewood *et al.* 2016*a*). Taken together, these findings suggest that sleep disturbance may moderate the relationship between pre-sleep entrapment and awakening levels of suicidal ideation.

To develop a deeper understanding of the inter-relationships between sleep disturbance, suicidal ideation, and entrapment, it is necessary to fulfill three important goals. First, research should include both objective and subjective assessments of key sleep parameters, which will counter retrospective recall biases. Second, research has yet to examine the bidirectional relationship between sleep disturbance and next-day suicidal ideation, and the ways in which daytime suicidal ideation relates to sleep the subsequent night. This is a surprising gap in the literature, given the wealth of research, which indicates that daytime emotions, such as loneliness and low mood, negatively impact sleep (Vandekerckhove & Cluydts, 2010; Kahn *et al.* 2013). Third, it is important to examine the inter-relationships between sleep disturbance, suicidal ideation, and entrapment, which will provide novel insights into the specific pathways by which sleep disturbance contributes to heightened suicidal ideation.

The current study addressed these three key goals using a repeated-sampling method known as ecological momentary assessment (EMA) or experience sampling methodology, which refers to the repeated, real-time sampling of individual experience (Larson & Csikszentmihalyi, 1983). The first hypothesis was that sleep disturbance would predict the increased severity of next-day suicidal ideation. The second hypothesis was that higher severity of daytime suicidal ideation would predict sleep disturbance the following night. The third hypothesis was that the relationship between pre-sleep entrapment and awakening levels of suicidal ideation would be moderated by poor sleep. For all three hypotheses, the key sleep parameters were both objective and subjective measures of total sleep time, sleep efficiency, and sleep onset latency, and a subjective measure of sleep quality.

Sleep disturbance (Sivertsen *et al.* 2009) and suicidal thoughts (Chesney *et al.* 2014) are commonly reported by people with diagnoses of different types of mental health problems. However, given the particularly high incidence of sleep disturbance reported by people with depression (Tsuno *et al.* 2005) and strong links between depression and suicide (Harris & Barraclough, 1997; Rihmer & Dome, 2016), this study focused on people who had experienced a major depressive episode(s). In addition, by focusing on a relatively homogeneous group in relation to mental health diagnosis, we sought to reduce the variability in, and therefore influence of, medication type and individual sleep architectural profiles. Similarly, in order to reduce between-subject variability in sleep patterns, the study focused on a restricted age group (18–65 years).

## Methods

### Participants

Participants were recruited from the National Health Service (NHS) mental health services, mental health charities, and via self-referral (May 2016 to March 2017). There were eight inclusion criteria: (1) experience of a major depressive episode(s) according to the Diagnostic and Statistical Manual of Mental Disorders (DSM-IV) and confirmed by the Structured Clinical Interview for DSM Disorders (SCID; First *et al.* 1997), (2) poor sleep quality as indicated by a score of >5 on the PSQI (Buysse *et al.* 1989), (3) self-reported suicidal thoughts and/or behaviors in the past month, (4) aged 18–65 years, (5) fluent in English, (6) no self-reported previous diagnosis of schizoaffective disorders or personality disorders, (7) no current symptoms of mania or hypomania, (8) no self-reported previous diagnosis of organic sleep disorders (i.e. narcolepsy, sleep breathing disorder, parasomnias). Participants received £30 compensation for their involvement in the study.

### Measures and procedure

The current study received ethical approval from the local NHS research ethics committee (ref: 16/NW/0226). There were four phases to this study which were (1) screening, (2) pre-EMA assessment and briefing, (3) 7-day EMA study, and (4) debriefing.

#### Screening

The SCID (First *et al.* 1997) was administered to confirm the experience(s) of major depressive episode(s) and no current symptoms of mania or hypomania. The SCID is extensively used as the criterion standard for depressive disorders (Lowe *et al.* 2004).

Screening for poor sleep quality was conducted with the PSQI, which is a 19-item questionnaire that measures sleep quality over the past month (Buysse *et al.* 1989). A cut-off score of >5 has demonstrated high sensitivity (89.6%) and specificity (86.5%) in differentiating individuals with good *v.* poor sleep quality (Buysse *et al.* 1989).

#### Pre-EMA assessment and briefing

First, participants completed demographical and questionnaire measures. Demographical information included the participant's gender, age, ethnicity, employment status, marital status, living circumstances, diagnosed mental health problems and current anti-depressant medication. Self-report measures of sleep (Sleep Condition Indicator; SCI), suicidal ideation (Beck Scale for Suicidal Ideation; BSSI), symptoms of depression (Beck Depression Inventory II; BDI-II), and anxiety (trait portion of the State Trait Anxiety Inventory; STAI-T) were also administered (see Table 1). Measures of depression and anxiety were included as possible confounding variables, given their strong inter-relationships with sleep disturbance and suicidal ideation (Harris & Barraclough, 1997; Tsuno *et al.* 2005). The BDI-II and STAI-T are recommended for examining mood within insomnia research (Buysse *et al.* 2006). To avoid any conflation in the effect size, a modified depressive symptoms score was calculated by subtracting the sleep item (Q16) and suicidal item (Q9) from the total BDI-II score (identified as MBDI-II). Details of the questionnaire measures are provided in Table 1.

**Table 1.** Descriptive statistics for pre-EMA measures of sleep, suicidal ideation, depression, and anxiety

Questionnaire measure	Score interpretation guidance	Median (IQR)	Range
Pittsburgh Sleep Quality Index (PSQI; Buysse *et al.* 1989)	Total scores can range from 0 to 21, with higher scores indicating worse sleep quality. A PSQI score of >5 indicates poor sleep quality	13 (10–16)	6–19
Sleep Condition Indicator (SCI; Espie *et al.* 2014)	Total scores can range from 0 to 32, and higher scores are indicative of better sleep. SCI score of ⩽16 corresponds with a probable diagnosis of insomnia disorder	8 (3−11)	0–26
Beck Scale for Suicidal Ideation (BSSI; Beck *et al.* 1988)	Total scores can range from 0 to 38, and higher scores suggest greater levels of suicidal thoughts and behaviors	15 (9–19)	0–28
Beck Depression Inventory II (BDI-II; Beck *et al.* 1996)	Total scores can range from 0 to 63, and higher scores indicate greater severity of depressive symptoms	38 (28–48)	14–56
State Trait Anxiety Inventory (STAI-T; Spielberger, 1983)	Higher scores specify greater trait anxiety, with a possible total score ranging from 20 to 80	65 (58–72)	33–78

Next, participants were given a detailed briefing of the EMA procedure to allow them to become familiar with the sleep diary used to collect subjective sleep data, and the PRO-Diary watch (CamNtech, Cambridge, UK), which collected objective sleep and EMA data. Daily sampling windows were agreed with participants, based on their habitual sleep and wake times with the aim of minimizing possible disruption of sleep due to diary alerts (Mulligan *et al.* 2016).

#### Seven-day EMA study procedure

All participants were asked to wear a PRO-Diary actigraph watch (CamNtech) on their non-dominant wrist for 24 h per day for 7 days. The watch contains an accelerometer unit that captures activity in 30-s epochs and can be used to estimate sleep and wake. Software sleep scoring algorithms were applied to extract three nightly objective sleep parameters: (1) sleep efficiency (proportion of time spent in bed asleep; %), (2) sleep onset latency (time taken to fall asleep; minutes), and (3) total sleep time (total time spent asleep according to actigraph wake/sleep categorization; hours). A previous study validated the accelerometer unit against PSG, to demonstrate its ability to provide reliable estimations of total sleep time, sleep onset latency, and sleep efficiency (Elbaz *et al.* 2012).

On waking each morning, participants completed the morning section of the expanded Consensus Sleep Diary, which consisted of 10 questions relating to their previous night's sleep (Carney *et al.* 2012). From these data, nightly subjective measures of sleep efficiency, sleep onset latency, and total sleep time (time spent sleeping from intention to sleep to final wake time) were calculated. Furthermore, subjective sleep quality was measured with the question ‘*How would you rate the quality of your sleep?*’, based on a five-point rating scale, ranging from very poor (1) to very good (5).

During the day, the watch alerted the participants at six quasi-random time points to complete the set of in-the-moment EMA items. To capture in-the-moment assessments across different time points, each individual's waking hours were split into six sampling windows, with the alert sounding at a random time within each window. All items were scored using a seven-point Likert scale, 1 = ‘*not at all*’ to 7 = ‘*very much so*’. Suicidal ideation was assessed by the item ‘*Right now I am feeling suicidal*’. Perceptions of entrapment were assessed by two items, which were designed to capture the desire to escape emotional pain ‘*Right now I want to escape my emotional pain*’ and perceptions that escape was prevented ‘*Right now I feel trapped*’. The mean value was calculated for the sum of the two items, to give the entrapment score.

#### Debriefing

At the end of the 7-day EMA study period, participants returned the PRO-Diary watch and sleep diary and were de-briefed in accordance with the guidance for conducting EMA studies with people experiencing mental health problems (Palmier-Claus *et al.* 2011). The researcher checked the data collected by the watch and sleep diary and clarified any ambiguities due to missing data (e.g. the watch had been removed due to participant swimming) or unclear responses (e.g. illegible handwriting on the sleep diary).

### Statistical analysis

Data analysis was conducted using STATA version 14. Grand medians, inter-quartile ranges (IQR), and ranges were calculated for all variables, as appropriate for the distribution of the data. The intra-class correlation coefficient was calculated for each variable to indicate the proportion of between-person and within-person variance. Data collected within this study were nested at three levels, namely, (1) participant, (2) day (7 days), and (3) EMA suicidal ideation and entrapment items (six times per day). Consequently, multilevel regression models were used to account for the nested nature of the data (Snijders & Bosker, 1999). Sleep variables were measured at the day level. Therefore, it was appropriate to calculate the daily mean of the suicidal ideation items to provide a day-level measure of suicidal ideation.

To test the first hypothesis that poor objective and subjective sleep parameters would predict next-day suicidal ideation, seven separate, two-level random intercept models were calculated. In each model, pre-EMA depression (MBDI-II) and anxiety (STAI-T) were included as control variables. To avoid multicollinearity, each of the seven sleep parameters (subjective total sleep time, objective total sleep time, subjective sleep efficiency, objective sleep efficiency, subjective sleep onset latency, objective sleep onset latency, and subjective sleep quality) were entered into separate regression models as predictor variables. Day-level suicidal ideation was entered as the outcome variable in each model.

To examine the second hypothesis, namely, that higher severity of daytime levels of suicidal ideation would predict poor objective and subjective sleep measures the subsequent night, a further seven separate, two-level random intercept models were calculated. Again, pre-EMA depression (MBDI-II) and anxiety (STAI-T) were entered as control variables, with day-level suicidal ideation as the predictor variable. Each model examined one of the seven specified sleep parameters as the outcome variable.

A final hypothesis examined a moderational pathway whereby the relationship between pre-sleep entrapment (at time point 6, i.e. the last measurement of the day) and awakening suicidal thoughts (at time point 1, i.e. the first measurement of the subsequent day) was moderated by poor sleep. First, the predictor and covariate variables were grand mean-centered. Seven separate two-level random intercept models were calculated to examine the possible moderation effects of each of the seven sleep parameters, which were acting as moderators. Pre-sleep entrapment was entered as the predictor variable, awakening suicidal thoughts as the outcome variable, and moderation was evaluated using the interaction between the predictor and moderator variables. Pre-EMA depression (MBDI-II) and anxiety (STAI-T) were entered as control variables.

Significance was evaluated at *p* < 0.05 for all analyses. Post-estimation residuals were plotted to assess normality of the distribution of outcome variables via histograms. A maximum likelihood estimation approach was taken, which allowed all available data to be included in the analysis.

## Results

### Participants

Fifty-four eligible participants were recruited into the study. An additional 23 people were screened but did not meet the full eligibility criteria for the study. Three individuals withdrew; two due to equipment failure problems and one withdrew on day 1, after experiencing anxiety due to the random timing of the sampling schedule. The final sample comprised 51 individuals. The majority of participants were recruited via self-referral from the community (*n* = 29), with the remaining participants recruited via NHS mental health services (*n* = 15) or mental health charities (*n* = 7). Sixty-seven percent of the sample were female (*n* = 34) and the overall mean age was 35.47 years (s.d. = 12.81, range 18–65 years). Most participants reported having previously made at least one suicide attempt (*n* = 36, 71%), chronic sleep problems lasting a year or more (*n* = 43, 84%), and currently taking anti-depressant medication (*n* = 34, 67%). Ninety-eight percent of participants (*n* = 50) reported suicidal ideation during the 7-day EMA sampling period (defined as at least one EMA suicidal ideation item score of >1). Descriptive statistics for the questionnaire measures are provided in Table 1, and descriptive statistics for the nightly objective and subjective sleep parameters, and EMA suicidal ideation and entrapment items are provided in Table 2.

**Table 2.** Descriptive statistics for daily measures of sleep and in-the-moment measures of suicidal ideation and entrapment

Variable	Data type	Median (IQR)	Range	Intra-class correlation coefficient
Total sleep time (min)	Subjective	420 (318–485)	0–805	0.33
	Objective	409 (358–452)	37–706	0.42
Sleep efficiency (%)	Subjective	81.37 (68.28–90.67)	0–100	0.35
	Objective	79.65 (71.4–85.5)	22.9–99.9	0.52
Sleep onset latency (min)	Subjective	30 (14–60)	0–380	0.35
	Objective	11 (1–37)	0–323	0.36
Sleep quality (1–5 very good)	Subjective	3 (2–3)	1–5	0.35
Suicidal ideation (1–7 high)	EMA	2 (1–4)	1–7	0.52
Entrapment (1–7 high)	EMA	4 (2.5–5.5)	1–7	0.58

### Adherence

Adherence to actigraphy monitoring, sleep diary, and suicidal ideation EMA items surpassed suggested minimum compliance levels of more than one-third complete entries per participant (Palmier-Claus *et al.* 2011). Overall, the sample completed 85% of suicidal ideation EMA items (1822/2147 items), 86% of entrapment EMA items (1839/2147 items), 91% of nights were monitored by actigraphy (326/357 nights), and 94% of nights were reported via the sleep diaries (334/357 nights).

### Does sleep disturbance predict higher levels of next-day suicidal ideation?

Table 3 summarizes the results of the seven, two-level, random intercept models calculated to examine the extent to which objective and subjective sleep measures predicted next-day suicidal ideation, adjusting for anxiety and depression symptom severity. Objective and subjective total sleep time and subjective sleep quality significantly predicted suicidal ideation the following day. Specifically, less objective and subjective total sleep time and poor sleep quality were associated with higher levels of next-day suicidal ideation. However, there were no significant associations between objective or subjective measures of sleep efficiency and next-day suicidal ideation.

**Table 3.** Effect of objective and subjective sleep parameters on next-day suicidal ideation (controlling for anxiety and depression symptom severity)

Predictor variable	Measure type	Unstandardized *β* coefficient	s.e.	Standardized *β* coefficient	*p*	95% Confidence intervals (CI)
Total sleep time (h)	Subjective	−0.0528	0.0233	−0.0801	0.024	−0.0984 to −0.0071
	Objective	−0.0760	0.0320	−0.0876	0.018	−0.1387 to −0.0133
Sleep efficiency (%)	Subjective	−0.0027	0.0028	−0.0344	0.346	−0.0082 to 0.0029
	Objective	−0.0088	0.0052	−0.0683	0.093	−0.0190 to 0.0015
Sleep onset latency (min)	Subjective	0.0006	0.0009	0.0250	0.491	−0.0011 to 0.0023
	Objective	0.0006	0.0012	0.0179	0.616	−0.0018 to 0.0031
Sleep quality (score 1–5)	Subjective	−0.1520	0.0504	−0.1093	0.003	−0.2508 to −0.0532

Outcome measure = suicidal ideation, scored on 1 (not at all) to 7 (very much so).

### Do high levels of daytime suicidal ideation predict sleep disturbance the subsequent night?

Table 4 shows results of seven two-level random intercept models calculated to test the reverse relationship, namely, that daytime levels of suicidal ideation predict objective and subjective sleep parameters the subsequent night. All analyses were non-significant.

**Table 4.** Effect of suicidal ideation on objective and subjective sleep parameters the following night (controlling for anxiety and depression symptom severity)

Outcome variable	Measure type	Unstandardized *β* coefficient	s.e.	Standardized *β* coefficient	*p*	95% Confidence intervals (CI)
Total sleep time (h)	Subjective	−0.0621	0.1023	−0.0408	0.544	−0.2627 to 0.1385
	Objective	−0.0046	0.0831	−0.0040	0.956	−0.1674 to 0.1583
Sleep efficiency (%)	Subjective	−0.7041	0.8728	−0.0543	0.420	−2.4149 to 1.0066
	Objective	0.3834	0.5746	0.0493	0.505	−0.7429 to 1.5096
Sleep onset latency (min)	Subjective	−1.9257	2.8661	−0.0464	0.502	−7.5431 to 3.6918
	Objective	−1.2015	2.0887	−0.0417	0.565	−5.2952 to 2.8923
Sleep quality (score 1–5)	Subjective	−0.0257	0.0499	−0.0358	0.606	−0.1235 to 0.0720

Predictor measure = suicidal ideation, scored on 1 (not at all) to 7 (very much so)

### Is the relationship between pre-sleep entrapment and awakening suicidal ideation moderated by poor sleep?

**Fig. 1.**
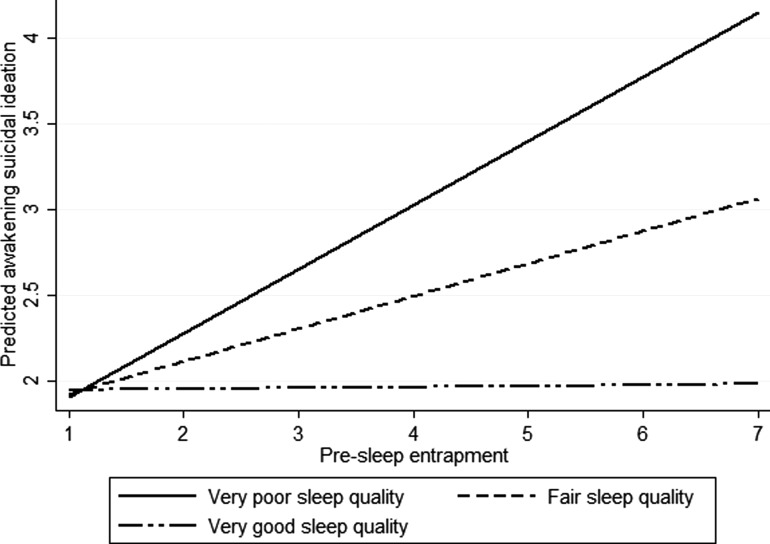
Moderation effects of sleep quality on the relationship between pre-sleep entrapment and awakening suicidal ideation.

A two-level multilevel model revealed a significant moderation effect of sleep quality, on the relationship between pre-sleep entrapment and awakening suicidal ideation, whilst controlling for pre-EMA depression and anxiety (*β* = −0.0909, 95% CI −0.1797 to −0.0022, *p* = 0.045). Figure 1 illustrates that when people reported poor sleep quality, higher pre-sleep entrapment was associated with increased awakening suicidal ideation. However, objective and subjective measures of total sleep time (objective *β* = 0.0076, 95% CI −0.0547 to 0.0700, *p* = 0.810; subjective *β* = −0.0025, 95% CI −0.0458 to 0.0408, *p* = 0.909), sleep efficiency (objective *β* = −0.0078, 95% CI −0.0183 to 0.0028, *p* = 0.148; subjective *β* = −0.0027, 95% CI −0.0078 to 0.0024, *p* = 0.304), or sleep onset latency (objective *β* = 0.0012, 95% CI −0.0014 to 0.0038, *p* = 0.370; subjective *β* = 0.0006, 95% CI −0.0013 to 0.0025, *p* = 0.526) did not moderate the association between pre-sleep entrapment and awakening suicidal ideation.

## Discussion

This study provides the first in-the-moment examination of relationships between objective and subjective sleep parameters, suicidal ideation, and entrapment. With respect to the first hypothesis, analyses revealed a unidirectional association between sleep disturbance and suicidal ideation. Specifically, shorter objective and subjective sleep duration and poor sleep quality predicted next-day suicidal ideation, after adjusting for anxiety and depression symptom severity. However, estimates of sleep efficiency and sleep onset latency did not predict next-day suicidal ideation. In relation to the second hypothesis, daytime suicidal ideation did not predict objective or subjective sleep parameters the following night. Finally, the third hypothesis investigated mechanistic pathways, which showed that only subjective sleep quality significantly moderated the association between pre-sleep entrapment and awakening levels of suicidal ideation.

These findings are in accord with the previous research, which has shown an association between self-reported short sleep duration and suicidal thoughts and behaviors (Gunnell *et al.* 2013; Winsler *et al.* 2015). However, contrary to the second hypothesis, severity of daytime suicidal ideation did not predict sleep disturbance the subsequent night. Here, our results extend the literature that has reported temporal precedence between sleep disturbance and suicidal ideation (Ribeiro *et al.* 2012; Zuromski *et al.* 2017), to show that poor sleep duration preceded increased severity of next-day suicidal ideation.

With regard to sleep quality, these results provide convergent evidence that subjective ratings of sleep quality are associated with suicidal ideation and behavior (Turvey *et al.* 2002; Bernert *et al.* 2014). Although estimates of sleep quality were based on individual's subjective perceptions, research has shown that ratings of sleep quality are positively associated with objective measures of slow wave sleep (SWS; Åkerstedt *et al.* 1997), which occurs during non-rapid eye movement (NREM) sleep. Measurement of sleep architecture was beyond the scope of the present study. However, there are corroborative findings from a recent PSG study, which reported that current suicidal ideation was associated with less NREM stage 4 sleep (Bernert *et al.* 2017*b*). SWS is thought to serve a restorative function, re-establishing synaptic homeostasis (Tononi & Cirelli, 2006); while insufficient SWS is associated with deficits in cognitive functioning (Lowe *et al.* 2017). Therefore, research should seek to examine the extent to which the relationship between poor sleep quality, less SWS, and suicidal ideation is mediated by cognitive functioning.

Recent research has sought to understand the psychological mechanisms that contribute to the relationship between sleep disturbance and suicidal thoughts and behaviors (for review see, Littlewood *et al.* 2017). The current study advances the cross-sectional literature by conducting the first temporal investigation of the inter-relationships between sleep disturbance, suicidal ideation, and entrapment. Perceptions of sleep quality were found to moderate the relationship between pre-sleep entrapment and awakening levels of suicidal ideation. Specifically, when people reported poor ratings of sleep quality, higher pre-sleep entrapment was associated with increased awakening levels of suicidal ideation. Conversely, when individuals had high pre-sleep entrapment but reported good sleep quality, they reported lower levels of awakening suicidal ideation. A note of caution is warranted here since the moderational effect of sleep quality yielded a *p* value of 0.045 and therefore replication is necessary with a larger sample size. However, these findings are consistent with a previous cross-sectional study, which reported a significant interaction effect between entrapment and symptoms of sleep disturbance (insomnia and nightmares) in predicting suicidal symptoms (Hochard *et al.* 2017). Moreover, a qualitative study found that sleep provided an escape from problems; and when individuals failed to escape via sleep, they described increased levels of suicidal thoughts (Littlewood *et al.* 2016*a*). Interestingly, findings in the current study appeared to be specific to subjective sleep quality, as opposed to the other measures of sleep efficiency, sleep duration, or sleep onset latency. Given that subjective ratings of sleep quality may be influenced by mood on awakening, this finding should be treated cautiously as preliminary evidence, which requires further empirical investigation.

The extent to which findings from this study generalize to other populations is an empirical question for future research. That said, the associations between sleep quality, sleep duration, and suicidal ideation have been reported across different populations, using different methodological designs (Krakow *et al.* 2011; Gunnell *et al.* 2013; Winsler *et al.* 2015). Furthermore, the moderation effect of sleep quality on the association between entrapment and suicidal ideation builds on the cross-sectional findings of Hochard *et al.* (2017), in which the sample largely comprised university students. Taken together, this provides preliminary evidence that the relationships reported in this study are not specific to those with depressive disorders. Contextually, it is important to note that participants in this study experienced prominent levels of clinical symptoms in that 98% reported suicidal ideation on one or more occasion during the study and 84% endured chronic sleep problems. This underscores the clinical importance of screening and treating sleep problems when working with the individuals who experience suicidal thoughts. Specifically, findings from the current study suggest that restoring healthy sleep duration and perceptions of sleep quality may be particularly effective in reducing the incidence of suicidal ideation. Furthermore, sleep may offer a temporary relief from waking problems, which may be helpful alongside interventions which seek to establish a more permanent resolution to the distressing problem. Promising pilot work shows that psychological interventions for sleep disturbance, such as cognitive behavioral therapy for insomnia and imagery rehearsal therapy for nightmares, may engender improvements in both sleep and suicidal thoughts (Trockel *et al.* 2015; Christensen *et al.* 2016; Ellis *et al.* 2017). However, it is unlikely that symptoms of sleep disturbance in isolation will offer clinical utility in the prediction and prevention of suicidal ideation. Indeed, it is improbable that any single risk factor will predict suicidal thoughts or behaviors with a high degree of accuracy (Franklin *et al.* 2017). Future research should examine the role of sleep duration and sleep quality in combination with a large range of other risk factors in predicting suicidal thoughts and behaviors (Franklin *et al.* 2017).

The current study has three key strengths. First, the micro-longitudinal EMA design is optimal for examining the experiences of individuals as they occur in real time. This is important when attempting to understand the factors which amplify and protect against mental health problems (Kleiman *et al.* 2017). In addition, because assessments were based on in-the-moment ratings, the EMA design did not suffer from any recall biases. Second, there was excellent adherence to the EMA schedule, completion of sleep diaries, and actigraphy monitoring. Compliance has been identified as a key issue when conducting EMA research (Palmier-Claus *et al.* 2011). Furthermore, the current study illustrates that individuals who were currently experiencing significant and severe symptoms of mental health problems should not be excluded from EMA-designed studies. Third, sampling schedules were customized to individual's habitual sleep/wake times to minimize possible disruption to their sleep (Mulligan *et al.* 2016). Surprisingly, this is a key departure from previous EMA protocols, which tend to follow seminal guidance by Larson & Csikszentmihalyi (1983) by standardizing the sampling schedules from 8:00 to 22:00 (e.g. Ben-Zeev *et al.* 2012). If this approach had been adopted in the current study, it is likely to have impacted on ecological validity, given that 49% (*n* = 25) of participants reported sleep/wake schedules which would have been interrupted by alerts sounding during the recommended sampling window.

Notwithstanding the contributions made by this study, three limitations should be noted. First, whilst actigraphy provided an objective estimate of sleep that can be easily administered in the natural environment, it cannot characterize sleep architecture which would require assessment via PSG (Ancoli-Israel *et al.* 2015). Second, in-the-moment suicidal ideation was assessed using a single item. Consequently, it is possible that this item alone may not provide a comprehensive measurement of suicidal thoughts in comparison with a multi-item questionnaire. Future research should consider additional items that seek to investigate different aspects of suicidal ideation, such as the intention to act on thoughts or ability to control intrusive thoughts (Van Spijker *et al.* 2014). Third, individuals with diagnosed organic sleep disorders, such as sleep apnea, were excluded from this study. It is possible that participants may have disorders that are yet to be diagnosed.

## Conclusion

The current study provides the first EMA examination of the temporal bidirectional associations between suicidal ideation and objective and subjective sleep parameters. In conducting a micro-longitudinal study, this advances the field by identifying specific parameters of sleep that predict next-day suicidal ideation, independent of anxiety or depression symptoms. Specifically, short objective and subjective sleep duration and poor perceptions of sleep quality predicted increased levels of suicidal ideation the following day. However, suicidal ideation during the day did not predict sleep the subsequent night. A moderation pathway indicated that poor sleep quality has the propensity to alter the strength of the association between pre-sleep entrapment and levels of suicidal ideation on awakening. These findings emphasize the importance of assessing sleep in individuals experiencing suicidal ideation and providing interventions targeted at boosting sleep duration and quality of sleep.
